# What Makes Antibodies Against G Protein-Coupled Receptors so Special? A Novel Concept to Understand Chronic Diseases

**DOI:** 10.3389/fimmu.2020.564526

**Published:** 2020-12-15

**Authors:** Gabriela Riemekasten, Frank Petersen, Harald Heidecke

**Affiliations:** ^1^ Clinic of Rheumatology and Clinical Immunology, University Hospital Schleswig-Holstein, University of Lübeck, Lübeck, Germany; ^2^ Research Center Borstel, Division of Pulmonary Immune Diseases, Member of the German Center for Lung Research (DZL), Borstel, Germany; ^3^ Research Center Borstel, Member of the German Center for Lung Research (DZL), Borstel, Germany; ^4^ Airway Research Center North (ARCN), Member of the German Center for Lung Research (DZL), Borstel, Germany; ^5^ CellTrend GmbH, Luckenwalde, Germany

**Keywords:** G protein-coupled receptors, anti-G protein-coupled receptor antibodies, immune cell homeostasis, precision medicine, angiotensin receptor 1

## Abstract

Expressions of G protein-coupled receptors (GPCR) on immune and tissue resident cells are the consequence of the cellular environment, which is highly variable. As discussed here, antibodies directed to GPCR (GPCR abs), their levels and correlations to other abs, serve as biomarkers for various diseases. They also could reflect the individual interplay between the environment and the immune system. Thus, GPCR abs could display pathogenic chronic conditions and could help to identify disease-related pathways. Moreover, by acting as ligands to their corresponding receptors, GPCR abs modulate autoimmune as well as non-autoimmune diseases. This article introduces GPCR abs as drivers for diseases by their capability to induce a specific signaling and by determining immune cell homeostasis. The identification of the individual GPCR ab function is challenging but might be pivotal in the comprehension of the aetiology of diseases. This, hopefully, will lead to the identification of novel therapeutic strategies. This article provides an overview about concepts and recent developments in research. Accordingly, GPCR abs could represent ideal candidates for precision medicine. Here, we introduce the term antibodiom to cover the network of abs with GPCR abs as prominent players.

## G Protein-Coupled Receptor Signature Reflects the Individual Response to Changing Environmental Conditions

Life requires recognition, response, and adaptation to changing conditions. Our ability to react to environmental stimuli, such as to taste and smell, indicates the need for a very sensitive system. G protein-coupled receptors (GPCRs) are crucial for these processes. They also regulate mood and behavior as well as our immune system (1–3). GPCRs are placed on cell membranes and are thus exposed to the variable and changing extracellular milieu. If not activated, GPCRs are thought to exist in a conformational equilibrium between active and inactive biophysical states (4). The flexible response to fluctuating environmental factors, a hallmark of GPCRs, is based on their capability to bind variable ligands, to change their conformations, to up- or down-regulate their membrane expression, and to complex with other proteins and receptors forming homo- or heterodimers. As consequence, GPCR functionally interact with a broad range of extracellular and intracellular proteins including signaling molecules as well as other membrane proteins. An example for close functional and local protein interactions starting with GPCR-mediated signaling is the activation of the GPCR dopamine 4 receptor stimulating platelet-derived growth factor receptor β (PDGFRβ) to inhibit the N-methyl-D-aspartate receptor (NMDAR) activity (5). However, even under physiological conditions, the environment is fluctuating in a certain range. Therefore, at each time point, a unique GPCR expression can be assumed to reflect the unique current micro and macro environment. Accordingly, GPCR expression and in sum the GPCR signature could image the cellular exposome. Functionally, changes in GPCR expression have consequences: They determine the cellular function *via* signaling as well as by affecting the binding capacity of their ligands.

Around 800 GPCRs have been described so far. Prominent examples of GPCRs are adrenergic receptors, cholinergic receptors, or proteinase activated receptors. GPCRs are widely expressed on immune cells.

## G Protein-Coupled Receptor Antiboides Often Correlate With Each Other’s and Thus Connect Different Proteins

Antibodies (abs) such as GPCR abs are a feature of the adaptive immune system and have been described first in vertebrates (2, 6). Based on the complex nature of GPCRs with three intra- and extracellular loops and intra-membranous domains, it is a challenge to develop test assays against the native structure. However, by using membrane extracts from cells overexpressing a specific GPCR, the levels of the abs can be sensitively measured by ELISAs. As recently shown by studying more than 30 abs to dopaminergic, serotoninergic, muscarinic, adrenergic, vascular, and immune receptors in humans, distinct correlations of the antibody levels have been identified. In addition, some GPCR abs also correlate with abs against growths factors and their receptors. The presence of a physiological antibody network of GPCR abs as central players, but also of other abs, is probably best reflected by using the term antibodiom. However, the correlations of the abs are slightly different between male and female healthy donors or between young and old persons. In general, old males revealed the highest number and strengths of correlations between various abs (2, 6). As example, abs against the angiotensin receptor type-1 (AT1R) strongly correlate with abs against the endothelin receptor type-A (ETAR). Abs against the complement C3a receptor 1 (C3AR1) correlate with F2R coagulation factor II receptor (PAR1) abs, and CXCR3 abs strongly correlate with CXCR4 abs (2). As shown before, both C3AR1 and F2R expressions are upregulated upon stimulation (7). In addition, CXCR3 as well as CXCR4 were shown to be strongly co-localized in the presence of inflammation (8). Therefore, and although the structural basis for these ab correlations is not known so far, it is very likely that they represent the expression status of their corresponding receptors and thus, indirectly, the exposome (9–11). In line with this, increased expressions of AT1R in peripheral blood mononuclear cells (PBMC), in the skin, as well as in the lung corresponded to increased AT1R ab levels in patients with systemic sclerosis (SSc), a severe autoimmune disease (12–17).

## G Protein-Coupled Receptor Antiboides Often Correlate With Each Others and Thus Connect Different Proteins Autoantibodies and the Antibodiom as a Long-Term Image of the Interplay Between the Cellular Exposome and the Individual Immune Response

As stated above, GPCRs can respond very fast to environmental factors. In contrast, changes in the adaptive immune system take much more time: GPCR abs are of the IgG subtype, which requires T cell help and class switches (18, 19). Our studies indicate that GPCR abs changed in a time frame of months or years (2). In other words, the GPCR abs are less sensitive as the GPCR to respond to acute changes in the environment. Therefore, GPCR abs represent most likely the chronic GPCR expression and activation status (GPCR signature). Accordingly, chronic cellular environmental factors (the chronic individual exposome) are reflected by the GPCR as well as the GPCR ab signature. This translation of the environment into the immunological memory *via* the antibodiom could be a novel mechanism for the interaction between the environment and the immune system. So far, studies on the interplay between GPCR abs and environmental conditions have just started.

Immune responses to antigens are variable between individuals based on different HLA haplotypes, which determines the antigen presentation to T cells. In addition, several genes involved in the very tight regulation of the immune response show polymorphisms. Therefore, the antibody generation including the immune response to GPCRs mirrors the individual immune system. In addition, the antibody network or the antibodiom reflects important individual processes, which makes it interesting for precision medicine.

## Increased and Decreased G Protein-Coupled Receptor Antibody Levels as Markers for Autoimmune And Non-Autoimmune Diseases

Increased abs against self-antigens are a feature of autoimmune diseases. Indeed, high GPCR abs are associated with several rheumatic autoimmune diseases such as with SSc, systemic lupus erythematosus (SLE), or primary Sjogren’s syndrome (2, 18–21). In SSc, AT1R abs as well as ETAR abs are increased and predict vascular complications such as pulmonary arterial hypertension as well as mortality and response to therapies (18, 19). Nevertheless, higher GPCR abs compared to healthy individuals are common in human pathophysiology and were also found in endocrinological diseases (Graves’ Disease or Hashimoto thyroiditis), in gynaecology (preeclampsia), cardiac diseases (heart transplantation, cardiomyopathy, chronic heart failure, orthostatic hypotension, and postural tachycardia syndrome), or in neurologic diseases such as in dementia, Alzheimer Disease, chronic fatigue syndrome (myalgic encephalomyelitis), or in complex regional pain syndromes (6, 13, 20–23).

In contrast, some diseases including autoimmune diseases are characterized by reduced levels of GPCR abs when compared to healthy donors. Thus, patients with giant cell arteritis have lower ETAR ab levels compared to healthy donors (24) and patients with granulomatosis with polyangiitis have reduced levels of antibodies against complement receptors (2). Furthermore, reduced levels of antibodies against ß1-adrenergic receptors are present in patients with acute coronary syndrome and are associated with a more severe disease particularly with a higher risk for early reinfarction and cardiovascular death in patients ≤ 60 years (25, 26). Lower abs against the thrombin receptor PAR-1 are associated with ovarian cancer and with high-grade carcinoma (2, 27). Finally, decreased levels of abs against the chemokine receptor CXCR3 and CXCR4 are found in patients with progressive interstitial lung disease (28, 29).

The presence of both increased as well as reduced GPCR ab levels further strengthen the idea of the presence of physiological levels and a balanced generation of autoantibodies in human physiology and pathophysiology.

## Correlations of Antibodies as Markers for Autoimmune and Non-Autoimmune Diseases

In addition, by studying the levels of multiple abs by ELISA tests in different diseases, characteristic alterations in the antibody correlations were identified, which differed in comparison to healthy donors (2, 13). In SSc, correlations changed between different GPCR abs, but also between GPCR abs and those directed to growth factors and their corresponding receptors (Tyrosine kinase receptors etc). In patients with SSc, new antibody correlations appeared between abs targeting AT1R, epidermal growth factor receptor (EGFR), and vascular endothelial growth receptors (VEGF-R1 and VEGF-R2) in comparison to heathy donors. Specific correlation changes were also observed in patients with Alzheimer Disease between neuronal receptors and growth factor receptors. Here, the dopaminergic and serotonergic ab pattern was associated with increased mortality, the cholinergic receptor pattern correlated with increased mood symptoms, and both of them were different to healthy controls (2, 3). Ovarian cancer was characterized by strong antibody correlations between different growth factors (2). Correlation changes could reflect changes in the GPCR expression pattern with hetero- and homodimerization and receptor co-expressions as well as interactions of GPCRs with other proteins, receptors, or signaling molecules. From an immunological point of view, changes in the antibody correlations could reflect epitope spreading and the emergence of novel so far hidden epitopes induced by acquired, exposome-induced changes in the GPCR signature. This break of tolerance leads to the generation of novel GPCR abs.

So far, it is not known whether these antibody correlations have an impact on the development of the different diseases. Only few functional studies exist analysing cross activation of the corresponding receptors by the correlating abs. Here, AT1R abs were shown to activate the ETAR and vice versa (30, 31) indicting functional consequences of ab correlations. However, ab levels as well as the ab correlations (the individual antibodiom) can serve as biomarkers for diseases.

## G Protein-Coupled Receptor Antibodies are Specific G Protein-Coupled Receptor Ligands Causing Unique Cellular Effects and Diseases

In the last decades, several GPCR abs were linked to the pathogenesis of very heterogenous diseases present in different medical disciplines. Sterin-Borda et al. described the effects of beta adrenergic abs as well as of other antibodies as potential drivers for Chagas disease (32). Wallukat et al. discovered functional beta-1 adrenergic receptor abs in patients with idiopathic dilated cardiomyopathy (33). AT1R abs are risk factors for rejection after solid organ transplantations and were shown to be drivers for non-HLA-dependent transplant rejections (21, 23, 34–36).

GPCR abs specifically bind to their corresponding receptors, which could have functional consequences as identified for several GPCR abs. As studied particularly in Graves’ Disease, neutral, stimulating, and blocking thyrotropin receptor (TSHR) abs have been described, which may compete for receptor binding with overlapping epitopes (21, 37–41). Here, stimulating TSHR abs cause hyperthyroidism and induction of a unique syndrome, which is different from sole hyperthyroidism initiated by the presence of increased peripheral hormones (41). *In vitro*, abs from SSc patients stimulated various cytokines (e.g. TGFß, IL-6, CCL18), chemokines (e.g. MCP1, IL-8), and adhesion molecules (VCAM-1) in cells such as in immune cells or in resident cells e.g. in microvascular dermal endothelial cells or in fibroblasts by signaling (18, 19, 22, 42) The reduction of the effects by AT1R and/or ETAR blockers indicates a contribution of anti-AT1R/ETAR abs. Indeed, our recent studies in mice proved the causal role of AT1R abs for interstitial lung disease, skin inflammation and skin fibrosis and, under certain circumstances, for obliterative vasculopathy (unpublished).

Thyrotropin receptor abs as well as AT1R abs are examples for stimulating and agonistic antibodies (6, 18, 41, 42). For most GPCR abs, the effects on their corresponding receptors still need to be deciphered.

Concerning the functional role of the antibodiom, specific effects of abs were also obtained by passive transfer of IgG fractions from SSc patients. Here, interstitial lung disease and vasculopathy, clinical signs of SSc, were induced (31). Although studied, no cytotoxic effects of the abs or complement activation have been observed. In addition, we recently have analysed the secretion of proteins upon stimulation of monocytes with purified IgG fractions from SSc patients. The secretome induced by IgG from patients was different to this from healthy donors and revealed associations with clinical symptoms (43). Other disease control studies are ongoing.

## G Protein-Coupled Receptor Antibodies Control Immune Cell Homeostasis and Avoid Systemic Immune Response to Acute Local Damage

**Figure 1:**
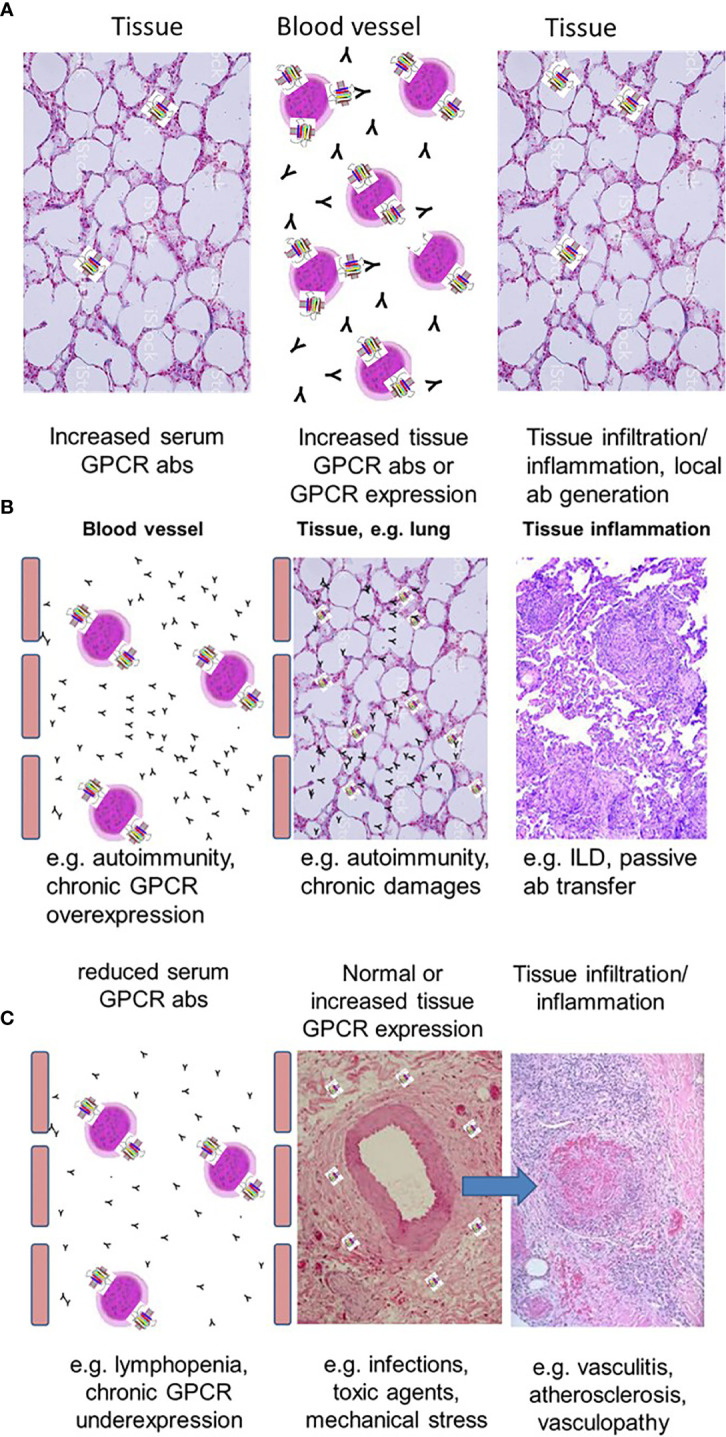
Concepts of altered GPCR abs in tissue inflammation. **(A)** Schematic presentation of a normal immune cell homeostasis with a tight balance between the levels of GPCR abs and GPCR expression of immune cells and tissue-resident cells. **(B)** Concept to explain tissue inflammation by either increased GPCR abs or increased tissue expression of GPCR. **(C)** Reduced GPCR ab levels could also cause tissue damage based on deficient competition to local tissue chemokines.

GPCRs are often expressed on immune cells whereas the expression of GPCR in tissues is very differently distributed. Some GPCRs are expressed mainly in a single tissue such as the thyrotropin receptor. Others such as AT1R are expressed in several organs (12, 14–16). The presence of a natural network of functional and regulatory abs could be important for several reasons: They stimulate immune cells to express cytokines and chemokines (17, 42). Therefore, GPCR abs may contribute to physiological blood levels of these mediators. In addition to the direct ab effects to the corresponding receptor *via* signaling, GPCR abs also attract cells expressing the corresponding receptors like other ligands. This was shown for abs against the endothelin receptor type-A (ETAR), AT1R, or the chemokine receptors CXCR3 and CXCR4 (2, 16, 17, 29). Therefore, both the abs as well as the GPCR ligands likely attract immune cells and, vice versa, the abs are attracted by the immune cells. This assumed equilibrium or steady state could be important for immune cell homeostasis between blood and tissues (**Figure 1A**). Accordingly, serum concentrations of ligands and abs could determine the threshold for tissue invasion to avoid an inadequate immune response to local tissue damage.

## Abnormal High G Protein-Coupled Receptor Antibodies and G Protein-Coupled Receptor Tissue Expression Cause Cell Invasion

According to the concept of a balance between GPCR abs and GPCR tissue expression, both the presence of abnormally high specific GPCR abs and abnormally high tissue expression of the corresponding receptor may cause immune cell invasion, which can be harmful (**Figure 1B**). Examples for the pathogenic effects of high GPCR abs are discussed before such as for Graves’ disease showing increased TSHR abs also leading to tissue inflammation (41). In addition, GPCR expression in the tissues can become upregulated e.g. by acute infections or in chronic conditions: Thus, increased lung AT1R expression was shown to be present in acute respiratory distress syndrome or acute lung injury induced by E. coli (44). Depending on the local expression level of AT1R (which probably corresponds to the degree of harm), AT1R abs as ligands will accumulate in the tissue with increased expression of AT1R, bind and activate AT1R (e.g. on fibroblasts) and will thus initiate signaling for the induction of chemokines. This could contribute to an adequate and balanced immune cell invasion, which is, under physiological conditions, necessary to couple with causes of harm.

In addition to microbes, exposure to diesel increased ETAR expression in the lungs of mice (45). In SSc, associations are present between the severity of interstitial lung disease (ILD) with the exposure to Benzol, which is another surrogate marker for traffic-induced air pollution (46). Here, endothelin receptor expressions are increased in the lungs (47).

Based on the nature of GPCR to respond to changing conditions, barrier organs such as lungs, the GI tract, or the skin might be more vulnerable to chronic environmental exposures (44, 45, 47). In addition, in barrier organs, the distance between blood and environment is low. Invasion of immune cells will certainly affect the normal function e.g. of the alveolar membrane.

## Possible Consequences of Local Immune Cell Invasion

Moreover, the accumulation of specific GPCR+ immune cells in the tissues and therefore, of the corresponding antigen, might also stimulate an immune response e.g. to AT1R leading to T cell help and the generation of AT1R+ B cells and plasma cells, which will locally produce AT1R abs. In our mouse model, we have partially seen germinal centre-like structures as well as ab binding (unpublished). So far, the specificity of the local tissue abs needs to be tested and this is an ongoing project in our studies. However, severe and uncontrolled tissue inflammation will result in a high consumption of cytokines such as of interleukin-2 (IL-2), which can lead to a local deficiency of regulatory T cells strongly requiring IL-2 (48). This could drive epitope spreading and autoimmunity to other autoantigens such as to down-stream signaling molecules of the activation cascade or to nuclear proteins as shown before in lupus (49). Theoretically, this could explain the generation of typical marker antibodies such as centromere abs in SSc. However, the hypothesis that GPCR abs initiate the immune response to the classical autoantigens needs to be proven by further studies.

## Abnormally Reduced G Protein-Coupled Receptor Antibodies as Biomarkers for Acute Damage and Possible Drivers for Vascular Damage

**Figure 2:**
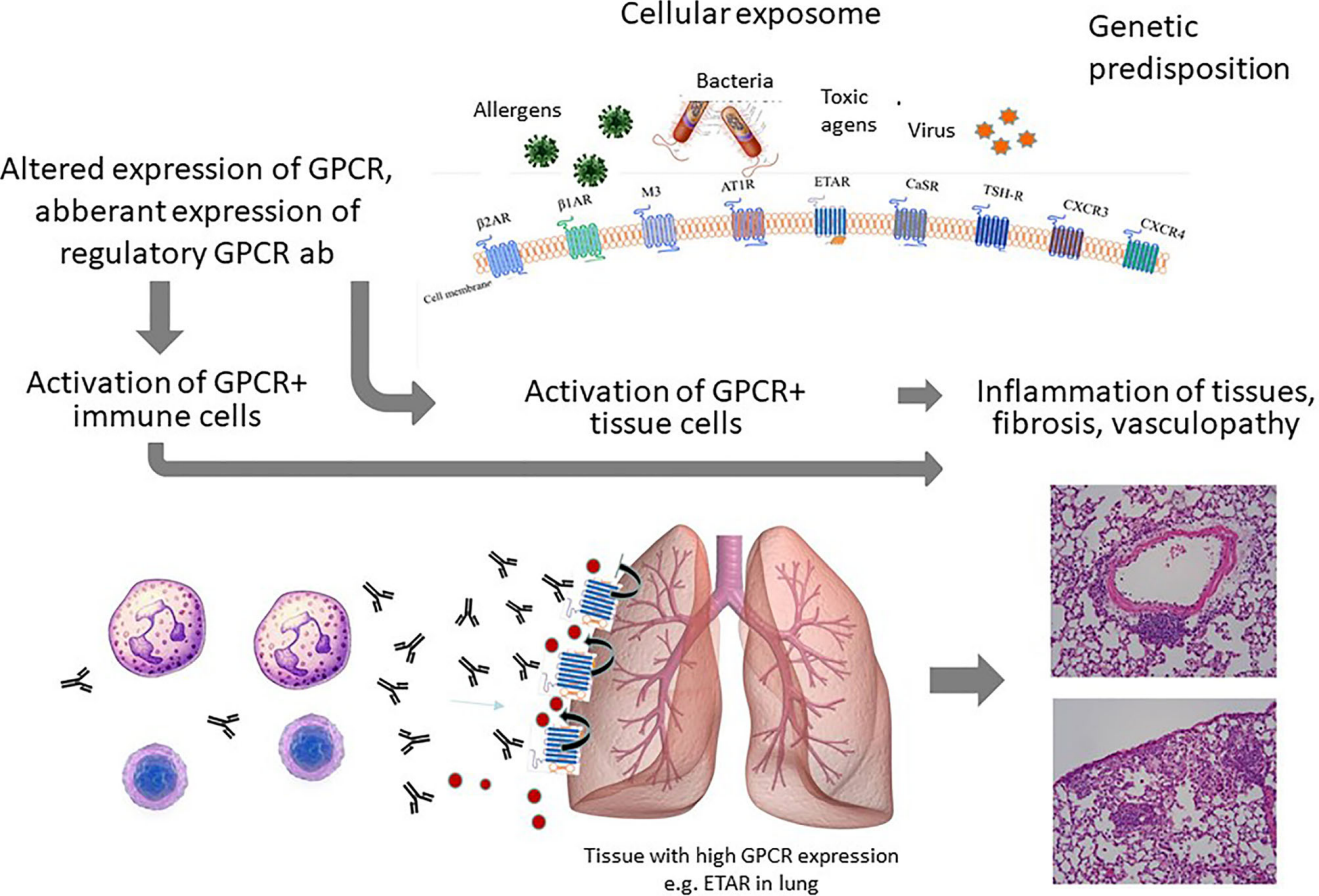
Summary of the concept: The chronic cellular exposome leads to an altered GPCR and GPCR ab signature. The ab effects on activation/migration of immune cells as well as tissue resident cells will lead to inflammation and vasculopathy as illustrated here for the lung.

In comparison to healthy donors, GPCR ab levels can also be reduced. As mentioned above, GPCR ab levels are in a tight balance with the general and chronic expression status of their corresponding GPCR, which can be up- or downregulated. Downregulation could result in a reduced GPCR ab response. In addition, some GPCRs are nearly exclusively expressed on immune cells. Examples for GPCRs mainly expressed by immune cells are the complement receptors 3 and 5 expressed on monocytes and macrophages. Another example is the chemokine receptor CXCR3, which is highly expressed on recently activated T cells. Under physiological conditions, the levels of the antibodies are low. Upon T cell activation, both CXCR3 ab levels as well as the serum concentrations of chemokines define the threshold for the migration of CXCR3+ T cells into the tissues by competing for receptor binding. Depending on the severity of damage, the number of tissue immune cells expressing CXCR3 could overreach the number of immune cells expressing CXCR3 in the blood. In those conditions, GPCR abs could become attracted to go into the tissues to bind their specific receptor or *via* cross-reactivity, other proteins. This concept could explain abnormally reduced ab levels in the blood as also shown for e.g. ß1 adrenergic receptor and ETAR abs in acute coronary syndrome or acute vasculitis flares, respectively (19, 20). Therefore, reduced ab levels could be a marker for a more acute tissue damage or of an acute flare (**Figure 1C**) and might contribute to vascular inflammations. This discussed scenario is still a hypothesis based on several associations, which needs to be proven. In addition, the identification of reduced GPCR ab levels by test assays is a challenge as well as the identification of the ab function for signaling. **Figure 2** provides a summary of the GPCR-GPCR ab interactions and the pathophysiological consequences.

## G Protein-Coupled Receptor Antibodies as Targets for Future Therapies

Unfortunately, GPCR abs are very resistant to immunosuppression as shown for AT1R abs in SSc patients (19) probably based on the ongoing presence of the exposome or the driving environmental factors, of the corresponding GPCR signature, and of the individual immune system. Therefore, reduction of abnormal high GPCR ab levels requires aggressive therapies such as autologous stem cell transplantation or combination of immunosuppressants (50–52). Both strategies are used in severe autoimmune diseases. To target the ab-mediated GPCR activation or ab-receptor-interaction more specifically, ab-neutralizing peptides or monoclonal idiotypic antibodies could be successful, which still needs to be shown. Although successful in animal models, small molecule inhibitors for the TSRH were not transferred into the clinic based on cost-effective alternatives (53). However, this could be different in other diseases such as in SSc or transplant rejection. Very recently, aptamers have been introduced as promising tools in nanomedicine. These small single-stranded DNA or RNA molecules can be used for the effective binding and removal of proteins such as antibodies. Aptamers specifically binding to some GPCR abs are currently investigated in animal studies showing promising data (54).

The presence of a functional and physiological network of abs and of a specific antibodiom is a theoretical basis for the use of intravenous or subcutaneous immunoglobulins in severe inflammatory diseases. The antibodies could reconstitute immune cell homeostasis. However; for long-term efficacy, very cost-intensive long-term and repetitive IVIG doses are required as shown for other diseases with established long-term IVIG therapies (49, 55). In addition, the ab-induced signaling could identify novel therapeutic targets as shown in a very recent manuscript identifying the AP1-pathway for the expression of the profibrotic cytokine CCL18 induced by abs (43). So far, current therapies target the binding and signaling of the natural ligands. The important role of GPCR abs in the pathogenesis of diseases and, therefore, as effector molecules, implicate to target the interaction of GPCR abs with their corresponding GPCR for future therapies.

## Further Potential Research Fields

GPCRs are also expressed on cancer cells. Therefore, GPCR abs could also be involved in the homeostasis of these cells and in the generation of metastases (56). In addition, they also may influence the reaction of the immune system to viral or bacterial agents, which emerge as an interesting field in the current pandemic.

## Conclusion

GPCR abs are special since they could reflect the interplay of the individual immune system with the individual exposome including the internal milieu. GPCR abs are together with the antibodiom biomarkers for autoimmune and non-autoimmune diseases. Based on their functions as ligands and regulators of the cell homeostasis, they can be used to identify further biomarkers, pathways, and therapeutic options. Specific GPCR abs cause and modulate diseases as shown so far for several GPCR abs. The identification of specific GPCR ab functions as well as the interplay between various antibodies could help to decipher diseases and their mechanisms. At best and hopefully, this provides a better approach to understand diseases. The future goal for the pharmaceutical industry should be to interfere with the interaction between abs, the physiological ligand, and their corresponding GPCR to develop novel drug candidates.

## Author Contributions

GR wrote the first draft, reviewed the manuscript, researched for this, wrote the last version, correspondence, ideas, and concept. FP reviewed the manuscript and graphic design. HH contributed ideas and concept, and reviewed the manuscript. All authors contributed to the article and approved the submitted version.

## Funding

This work was supported by the Deutsche Forschungsgemeinschaft (DFG), the DFG-founded excellence cluster Precision Medicine in Inflammation (PMI, project TI4), GRK1727 “Modulation of Autoimmunity”, by the DFG single project RI 1056 11-1/3, by the Eppenauer Gutzeit foundation, and by funding from the German Center for Lung Research (DZL).

## Conflict of Interest

HH is the director of the company CellTrend, developing and selling ab assays for the detection of a large number of GPCR abs. GR is an advisor for CellTrend. She received partial honorary for her advice as well as ELISA plates and membrane extracts for research purposes. CellTrend is a company producing tests for the detection of autoantibodies such as against AT1R, ETAR, and others. All the tests were used by the authors. GR received fees to be a member of the advisory board for CellTrend.
